# Secretome Analysis of Skeletal Myogenesis Using SILAC and Shotgun Proteomics

**DOI:** 10.1155/2011/329467

**Published:** 2011-03-29

**Authors:** C. Y. X'avia Chan, John C. McDermott, K. W. Michael Siu

**Affiliations:** ^1^Department of Biology, York University, 4700 Keele Street, Toronto, ON, Canada M3J 1P3; ^2^Centre for Research in Mass Spectrometry (CRMS), York University, 4700 Keele Street, Toronto, ON, Canada M3J 1P3; ^3^Muscle Health Research Centre (MHRC), York University, 4700 Keele Street, Toronto, ON, Canada M3J 1P3; ^4^Centre for Research in Biomolecular Interactions (CRBI), York University, 4700 Keele Street, Toronto, ON, Canada M3J 1P3; ^5^Department of Chemistry, York University, 4700 Keele Street, Toronto, ON, Canada M3J 1P3

## Abstract

Myogenesis, the formation of skeletal muscle, is a multistep event that commences with myoblast proliferation, followed by cell-cycle arrest, and finally the formation of multinucleated myotubes via fusion of mononucleated myoblasts. Each step is orchestrated by well-documented intracellular factors, such as cytoplasmic signalling molecules and nuclear transcription factors. Regardless, the key step in getting a more comprehensive understanding of the regulation of myogenesis is to explore the extracellular factors that are capable of eliciting the downstream intracellular factors. This could further provide valuable insight into the acute cellular response to extrinsic cues in maintaining normal muscle development. In this paper, we survey the intracellular factors that respond to extracellular cues that are responsible for the cascades of events during myogenesis: myoblast proliferation, cell-cycle arrest of myoblasts, and differentiation of myoblasts into myotubes. This focus on extracellular perspective of muscle development illustrates our mass spectrometry-based proteomic approaches to identify differentially expressed secreted factors during skeletal myogenesis.

## 1. Introduction

Myogenesis, the formation of skeletal muscle, has been recognized as a hierarchical cellular event, commencing with myogenic lineage specification and followed by iterative proliferation of the muscle precursor cells called myoblasts in which cell-cell contact is initiated. This triggers withdrawal of myoblasts from the proliferation cycle (i.e., cell-cycle arrest) and in turn switches on the differentiation program in which mononucleated myoblasts are fused to each other and give rise to multinucleated myotubes (i.e., building blocks for contractile muscle fibres in the mature animal). Each step is orchestrated by groups of intracellular factors, such as cytoplasmic signalling molecules and nuclear transcription factors, which are described in further detail below. 

### 1.1. Myogenic Lineage Specification

**Figure 1:**
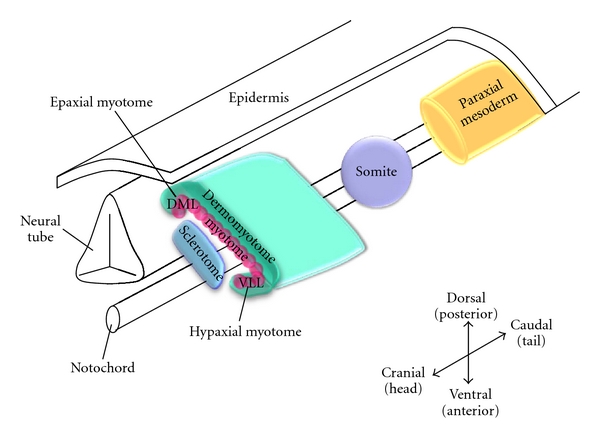
Myogenic lineage specification. Dorsal medial lip and ventral lateral lip were denoted as DML and VLL, respectively. Redrawn from Buckingham et al. [6].

Skeletal muscle originates from the paraxial mesoderm, epithelialization and segmentation of which gives rise to the somites in a cranio-caudal manner (i.e., somites are generated and specified from head to tail) (Figure 1). Various compartments of the somite are committed to distinct cell lineages: myotome (muscle), dermatome (skin), and sclerotome (bone and cartilage), according to their relative orientations to the surrounding tissue, such as ectoderm, neural tube, notochord, and lateral mesoderm [1]. The ventral medial portion of the somite is specified as the sclerotome, whereas the double-layered structure remaining is called the dermomyotome which gives rise to the dermatome and myotome. The latter is subdivided into two compartments: dorsal medial lip (DML) and ventral lateral lip (VLL). The former compartment gives rise to the epaxial myotome that becomes the back muscle, whereas the latter gives the hypaxial myotome that generates the muscles of the body wall, limbs, and tongue [2–5]. 

### 1.2. Myoblast Proliferation with Simultaneous Repression of Muscle Differentiation

**Figure 2:**
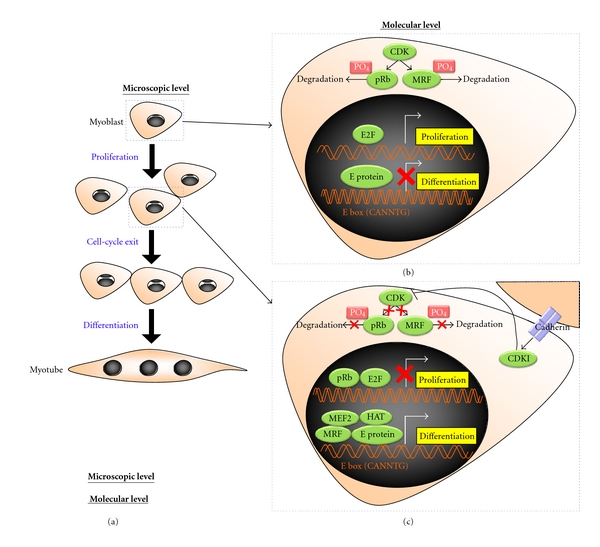
Skeletal muscle differentiation at the microscopic and molecular level. (a) During myogenesis, mononucleated myoblast proliferate, followed by cell-cycle exit, and fusion to form multinucleated myotube; (b) during proliferation, at the molecular level, active CDK could trigger myoblast proliferation by phosphorylating and subjecting pRb to degradation, in which E2F transcription factor is free from the inhibitory effect of pRb and elicits the proliferation of myoblasts. Simultaneously, CDK can also block myoblasts from differentiation via the phosphorylation-induced degradation of MRF. As a consequence, E protein by itself cannot drive the differentiation program; (c) upon cell-cell contact, m-cadherin is activated, by which CDKI is induced. This in turn inhibits CDK from phosphorylating its downstream substrates: pRb and MRF. Hence, both pRb and MRF are exempted from degradation, in which the former can withdraw the myoblasts from the cell cycle by inhibiting E2F transcription factor from activating the proliferation-associated events, whereas the latter complexes with E protein, myogenic co-activator MEF2, and the chromatin remodeling molecule HATs, in an effort to evoke the differentiation program of myoblasts synergistically. Phosphate groups were indicated as “PO_4_”.

After the primary wave of myoblasts is generated from the somite, they enter the cell cycle and undergo iterative propagation to expand the cell population, eventually cell-cell contact occurs. This step has been shown to be essential to withdraw the myoblasts from the proliferation cycle and initiate the differentiation program (Figure 2(a)) [7–9]. Thus, the proliferation and differentiation of myoblasts are mutually exclusive events; the tipping point between the two is governed by a master regulator: the retinoblastoma protein (pRb) [10–12].

During proliferation, cyclin/cyclin-dependent kinases (CDKs), such as cyclin D/cdk4, cyclin D/cdk6, cyclin E/cdk2, and cyclin A/cdk2, are active. These kinases phosphorylate pRb, holding it inactive [13–18]. As a result, pRb is unable to bind to the E2F transcription factor complex and inhibit its activation of downstream proliferation-associated cellular events, including chromosome segregation, mitotic spindle formation, and chromatin remodelling [19] (Figure 2(b)). 

Notably, the differentiation of these myoblasts is critically dependent upon a family of myogenic transcription factors: the myogenic regulatory factors (MRFs), including myogenic differentiation factor (MyoD) [20, 21] and myogenic factor 5 (Myf5) [22, 23]. The MRFs confer on the myoblasts a potent ability to differentiate. By contrast, mitogenic myoblasts may be prohibited from differentiation by myogenic repressors, including Id [24, 25], twist [26–28], MyoR [29, 30], Mist 1 [31], and I-mf [32]. In the absence of myogenic repressors, MRFs, which are members of the class II basic helix-loop-helix (bHLH) superfamily, can dimerize with members of the class I bHLH family, the E proteins. The E protein: MRF heterodimer thus resulted recognizes and binds to the consensus DNA sequence (CANNTG) named the E-box, which lies upstream of most muscle-specific genes, for example, the myosin heavy chain and muscle creatine kinase [33]. Conversely, in the presence of myogenic repressors, the dimerization between MRF and the E protein inside the nucleus is negated either by (1) competitive binding to MRFs or the E proteins by means of Id, twist, MyoR, and Mist 1, or (2) sequestering MRFs in the cytoplasm by means of I-mf. Additional control can come via other interactions, including those of pRb and CDKs which can also phosphorylate MRFs and subject them to degradation [34–36] (Figure 2(b)). The initial repression of muscle differentiation is essential for ensuring a sufficiently large number of myoblasts are attained prior to differentiation to populate the vast amount of skeletal musculature in the metazoan species. 

### 1.3. Cell-Cycle Arrest of Myoblasts with Simultaneous Activation of Muscle Differentiation

Under growth conditions, myoblasts proliferate until they reach confluency and cell-cell contact provokes growth arrest. The switch between cell-cell contact and cell-cycle arrest is mediated by transmembrane proteins, such as m-cadherin [37–42]. Upon cell-cell contact, m-cadherin is activated and induces CDK inhibitors (CDKIs), for example, p21 and p57 [43, 44]. As the name suggests, CDKIs inhibit CDK from phosphorylating its respective substrates, such as pRb and MRF [45, 46]. As a result, both pRb and MyoD are spared from degradation. The corollary to that is twofold: (1) nonphosphorylated pRb can bind and inhibit E2F from activating the downstream proliferation events, by which cell-cycle arrest of myoblasts is achieved [47, 48]; (2) nonphosphorylated MyoD can dimerize with the E protein and cooperatively bind to the E box to activate the expression of muscle-specific gene, thus triggering the differentiation program. Furthermore, with the recruitment of myogenic coactivators, such as myocyte enhancer factor 2 (MEF2) [49–52] as well as the chromatin remodelling factors, the histone acetyltransferases (HATs), for example, p300 and p300/CBP-associated factor (PCAF) [53–62], the differentiation program is initiated (Figure 2(c)). In addition, activated cadherin interacts and triggers a cell adhesion molecule of the Ig superfamily called CAM-related/downregulated by oncogenes (CDO) [63, 64]. The CDO complex promotes myogenesis by activating the p38 MAPK signalling pathway [65–68], which is a well-known promyogenic signal acting at various steps [69–71]. p38, for example, enhances the activity of MyoD [72], and its co-activator MEF2 [73], favouring MyoD/E protein heterodimerization by phosphorylating E protein [74], recruiting SWI-SNF chromatin-remodelling complex to the promoter of muscle-specific genes to enhance accessibility to transcriptional regulators required for subsequent gene expression [75]. Intriguingly, CDO is a target of MyoD, establishing positive feedback loop which reinforces the muscle differentiation program [64, 76]. 

### 1.4. From Intra- to Extracellular Perspective of Myogenesis

Irrespective of well-documented intracellular factors entailed in myogenesis, the key step in developing a more comprehensive picture of the regulation of muscle development is to investigate the extracellular factors that prime these downstream intracellular events. This, in turn, may provide valuable insight into the acute cellular response as a result of extrinsic cues in normal muscle development and regeneration. Intriguingly, the effects exerted by the “conditioned” media (CM) on the development of muscle cells have been documented some time ago [77, 78], illustrating the phenomena that myogenic cells modify their own extracellular milieu by secreting factors that exert autocrine and paracrine effects on the differentiation program. Furthermore, the skeletal muscle has been recognized as the largest endocrine organ in humans for secreting extracellular factors, the myokines that orchestrate muscle development in an autocrine fashion [79, 80]. Apart from the well-known myokines, such as members of the insulin-like growth factor-1 (IGF1) [81–90] and transforming growth factor (TGF) families [91–99], which have potent, but opposing effects on myogenesis, there were individual studies investigating other myokines, such as plasminogen activator [100], collagenase [101], decorin [102], glial growth factor [103], neurocrescin [104], meltrin alpha [105], musculin [79, 106], interleukin-1 beta [107], interleukin-7 [108], ADAMTS-like 2 [109], follistatin-like 1 [110], secreted protein acidic and rich in cysteine (SPARC) [111–113]. To make progress on the characterization of the “secretome” in an unbiased manner, we implemented an initial mass spectrometry-based proteomics study to identify secreted proteins in the mouse skeletal muscle cell line C2C12 [114]. Furthermore, a more quantitative approach using stable-isotope labelling by amino acids in cell culture (SILAC) in conjunction with online reverse phase liquid chromatography tandem mass spectrometry (RPLC-MS/MS), has now been implemented to identify differentially expressed secreted proteins during myogenesis. 

## 2. Workflow of SILAC Quantification

In differential proteomics, stable-isotope labelling, for example, ^2^H versus ^1^H, ^13^C versus ^12^C, and ^15^N versus ^14^N, is employed to introduce a signature mass difference between the samples of interest (e.g., treatment versus control). After enzymatic protein digest, the ratios of the labelled peptide peak intensities reveal the relative protein expression. There are two general ways to introduce the stable-isotope label into the sample: (1) chemical labelling, typically achieved via the isotope-coded affinity tag (ICAT) or the isobaric tag for relative and absolute quantitation (iTRAQ); (2) metabolic labelling, conveniently performed via SILAC. ICAT targets the sulfhydryl group on the cysteine residue [115], whereas iTRAQ modifies the amino group on the N-terminus and the lysine residue [116]. For SILAC, stable-isotope labelled amino acids are metabolically incorporated into the living cells as they grow. Irrespective of the labelling methodology, the tagged samples are then combined and processed as one in subsequent treatment, separation, and analysis. This minimizes the impact of nonquantitative recovery of the proteins and peptides in these steps on the accuracy of the quantification [117, 118]. 

In recent years, SILAC has been widely applied to various biological models and cell types, including immune B cells [119], fibroblasts [120], neuronal cells [121], blood cells [122], lung cells [123], chondrocytes [124], prostate cancer [125], ovarian cancer [126], liver cancer [127, 128], breast cancer [129, 130], esophageal cancer [131, 132], and embryonic stem cells [133–135]. In addition, it has also been successfully implemented in tissues [136, 137] and living organisms [122, 138–140].

**Figure 3:**
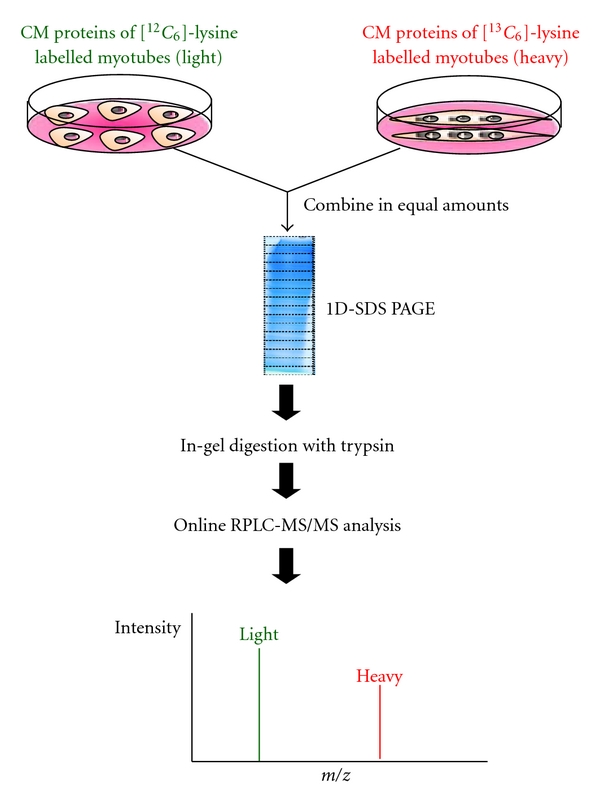
The workflow of using SILAC to identify differentially expressed secreted factors during skeletal myogenesis.

We employed SILAC labelling in an attempt to identify differentially expressed secreted factors at the myotube- versus myoblast-stage (i.e., differentiation versus proliferation) in C2C12 cells. As illustrated in Figure 3, CM proteins derived from [^12^C_6_]-lysine labelled myoblasts (light) and [^13^C_6_]-lysine labelled myotubes (heavy) were mixed in equal amounts and subjected to one-dimensional gel electrophoresis (1D-SDS PAGE), followed by trypsin digestion. The resulting tryptic peptides were analyzed by online RPLC-MS/MS. The ratio of the heavy- versus light-labelled peptide peak intensities in the MS mass spectrum mirrored the relative expression level of that particular protein during myogenesis. 

## 3. Implications of the Secreted Proteins Identified in Myogenesis

As previously discussed, myogenesis is a multistep process, beginning with myogenic lineage specification, followed by cell proliferation, cell-cycle arrest, and ultimately the differentiation of myoblasts into myotubes. We postulated that each of these steps is regulated by secreted factor(s). According to our preliminary data, novel secreted proteins, such as osteoglycin (OGN), peroxiredoxin 1 (Prx1), and cytokine-induced apoptosis inhibitor 1 (CIAPIN1), were identified as differentially expressed proteins. Their respective role(s) in myogenesis were proposed as follows. 

### 3.1. OGN

OGN is also known as mimecan. It belongs to the small leucine-rich repeat proteoglycan (SLRP) family of proteins [141–147]. This protein was found to be essential in maintaining the integrity of the extracellular matrix (ECM) of the cornea [148, 149] and the vascular smooth muscle [150, 151] by inhibiting the ECM-cleaving enzyme gelatinase [152]. This anti-ECM cleaving property contributed to OGN's tumour suppressor role in hepatocarcinoma cells by attenuating tumour cell migration [153]. Given OGN's differential expression in myogenesis, we hypothesized that OGN may play an inhibitory role by hindering myoblast migration and the subsequent cell-cell contact. As result, cell-cycle arrest is inhibited and hence the muscle differentiation program is sabotaged. Interestingly, the E box has been identified in the promoter region of OGN [154]. This projects a compelling regulation mechanism of OGN during myogenesis in which binding of the MRF and E protein heterodimer to the E box may function as a docking site to recruit a chromatin remodelling molecule, such as histone deacetyltransferases (HDACs); as consequence, the transcription and subsequent expression of OGN decrease. 

Furthermore, OGN may also play a role in myogenic lineage commitment, where the protein was initially identified as a bone-inductive factor [155–159]. Intriguingly, we have demonstrated the possibility that C2C12 myoblasts could be recommitted to the osteoblast lineage by overexpressing a bone-inductive gene called menin1 [160]. With this taken into account, it is tempting for us to speculate a plausible link between OGN and menin1 in which downregulation of OGN may be essential in directing the myoblasts to myogenic lineage. 

### 3.2. Prx1

Prx1, also known as Pag [161] or MSP23 [162], belongs to the antioxidant protein family for cellular defence against reactive oxygen species (ROS) [163]. Prx1 was revealed to be upregulated in various cancer types, such as oral cancer [164], lung cancer [165–172], pancreatic cancer [173], and esophageal cancer [174]. Expression level of Prx1 was shown to positively correlate with cancer progression; knocking down Prx1 not only attenuated malignancy, but also sensitized the cancer cells to chemotherapy and improved survival [175–177]. Given the role of Prx1 as a prosurvival factor by blocking apoptosis signal-regulating kinase (ASK)- induced cell death [178–180], we hypothesized that Prx1 may function as a mitogen that promotes the proliferation of myoblasts. As proliferation and differentiation are mutually exclusive events, the down-regulation of Prx1 (unpublished data) may be essential for the withdrawal of myoblasts from the proliferation cycle and subsequent differentiation. 

### 3.3. CIAPIN1

CIAPIN1 has been characterized as an antiproliferation molecule in cell division and angiogenesis [181–183]. CIAPIN1 was shown to be a suppressor of various cancers, for instance gastric cancer [184], renal carcinoma [185], esophageal cancer [186], and colorectal cancer [187]. The antiproliferation effect of CIAPIN1 was found to be mediated by upregulating CDKI, which in turn allows pRb to inhibit the E2F transcription factor from activating downstream proliferation events; as a result, cell-cycle arrest prevails [185, 188]. We postulated that CIAPIN1 may function as a positive regulator of myogenesis, in which the upregulation of CIAPIN1 (unpublished data) may be essential in triggering cell-cycle arrest of myoblasts for subsequent differentiation to take place. 

## 4. Conclusion

**Figure 4:**
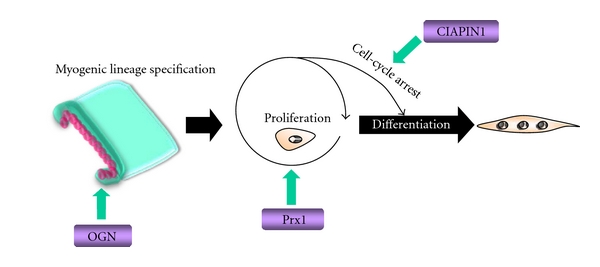
Overview of the implications of OGN, Prx1, and CIAPIN1 in myogenesis.

We have demonstrated the fidelity of applying SILAC to identify secreted factors during skeletal myogenesis in an unbiased proteomics approach. OGN, Prx1, and CIAPIN1 were identified as novel differentially expressed extracellular factors that are proposed to play a role in the myogenic program (Figure 4). Based on the findings of this “discovery” approach, gain and loss of function studies are now in progress to further dissect these proteins' individual and combinatorial roles in myogenesis. The identification of secretome factors that regulate myogenesis will enhance our knowledge of extracellular regulation of differentiation as well as identify biomarkers of potential therapeutic value in muscle regeneration and stem cell programming. 

## Abbreviations

1D-SDS PAGE: One-dimensional gel electrophoresis
ASK: Apoptosis signal-regulating kinase
bHLH: Basic helix-loop-helix
CDKIs: CDK inhibitors
CDKs: Cyclin-dependent kinases
CDO: CAM-related/downregulated by oncogenes
CIAPIN1: Cytokine-induced apoptosis inhibitor 1
CM: Conditioned media
DML: Dorsal medial lip
ECM: Extracellular matrix
HATs: Histone acetyltransferases
HDACs: Histone deacetyltransferases
ICAT: Isotope-coded affinity tag
IGF1: Insulin-like growth factor-1
iTRAQ: Isobaric tag for relative and absolute quantitation
MEF2: Myocyte enhancer factor 2
MRFs: Myogenic regulatory factors
Myf5: Myogenic factor 5
MyoD: Myogenic differentiation factor
OGN: Osteoglycin
PCAF: p300/CBP-associated factor
pRb: Retinoblastoma protein
Prx1: Peroxiredoxin 1
ROS: Reactive oxygen species
RPLC-MS/MS: Reversed phase liquid chromatography tandem mass spectrometry
SILAC: Stable isotope labelling by amino acids in cell culture
SLRP: Small leucine-rich repeat proteoglycan
SPARC: Secreted protein acidic and rich in cysteine
TGF: Transforming growth factor
VLL: Ventral lateral lip.
